# Frequency of Pediatric Acute Respiratory Distress Syndrome Based on Oxygen Saturation Index in Pediatric Intensive Care Unit of a Developing Country

**DOI:** 10.7759/cureus.6444

**Published:** 2019-12-22

**Authors:** Rahim Ahmed, Asim Azim, Azam Nangialay, Anwar Haque, Humaira Jurair

**Affiliations:** 1 Pediatric Intensive Care Unit (PICU), The Indus Hospital, Karachi, PAK; 2 Pediatrics, Aga Khan University Hospital, Karachi, PAK; 3 Pediatrics, The Indus Hospital, Karachi, PAK; 4 Pediatric Intensive Care Unit (PICU), Aga Khan University Hospital, Karachi, PAK

**Keywords:** pediatric acute respiratory distress syndrome, oxygen saturation index, picu

## Abstract

Objectives

To determine the frequency of pediatric acute respiratory distress syndrome based on oxygen saturation index in pediatric intensive care unit of a developing country.

Methods

We conducted a retrospective study of all children admitted in pediatric intensive care unit (PICU) of Aga Khan University Hospital, Karachi from July 2017 to June 2018 with respiratory rate >40 breaths/minute, shortness of breath, and bluish discoloration of skin and mucous membranes. The diagnosis of acute respiratory distress syndrome (ARDS) was made on the basis of standard operational definitions as mentioned (fulfilling criteria for ARDS).

Results

During the one-year study period 150 patients with age range of one month to 16 years were admitted fulfilling the inclusion criteria. Mean age was 38.27 ± 53.13 months, and 92 (61.33%) were male with male to female ratio of 1.6:1. Mean duration of symptoms was 1.23 ± 0.42 days. Frequency of pediatric acute respiratory distress syndrome using oxygen saturation index admitted in a pediatric ICU was 23 (15.33%) patients.

Conclusion

This study has shown that the frequency of pediatric acute respiratory distress syndrome is quite high.

## Introduction

Acute respiratory distress syndrome (ARDS), evident by persistent hypoxemia, decrease respiratory system compliance and nonhydrostatic pulmonary edema, is a serious and complex clinical problem with a high morbidity, mortality and financial cost, especially in a resource-limited situation. The reported morbidity and mortality ranges from 14% to 61% in developed countries and even high in developing countries [1,2]. It involves a series of events following acute lung injury and can be triggered by a variety of insults, including pneumonia, sepsis, aspiration, shock, burns and traumatic injury, all results in inflammation and increased vascular permeability leading to pulmonary edema [3].

ARDS was defined in different times and due to lack of universal accepted definition till now has led to a series of practical problems for a definitive diagnostic guidelines and hence therapeutic strategies. ARDS was previously diagnosed on the basis of American-European consensus conference definition, changed to Berlin definition in 2012, by European intensive medicine society [4,5]. Recently, the pediatric acute lung injury consensus conference group described the latest definition of pARDS based on oxygen saturation (OI) and oxygen saturation index (OSI) [6].

It is not uncommon diagnosis in pediatrics intensive care units (PICUs) and estimated prevalence ranges from 1.4% to 2.7% of all PICU's admissions [1,3]. The reported data regarding incidence of pARDS varies from study to study and time to time depending on the patient population targeted. The epidemiological data available is mostly from the developed countries and there is scant data on incidence of pARDS in developing countries [3,7]. A recent study from India reported pARDS incidence from 8.5 to 27 cases per 1000 PICU admissions [2]. Another study from Iran reports incidence from 5 to 10% of total PICU admissions [8]. We found no available data on incidence of pARDS in Pakistan after a thorough literature search.

Pulse oximetry is the most commonly utilized technique to monitor oxygenation, noninvasive, and safe. Pulse oximetry prevents arterial blood sampling and curtails cost for arterial blood gas analysis [9]. The oxygenation saturation index (OSI) and oxygen saturation (SF) ratio used as an oxygenation metrics instead of oxygenation index (OI) [Fraction of inspired oxygen concentration x mean airway pressure / pulse oximetry saturation] or PF ratio [partial pressure of oxygen/fraction of inspired oxygen concentration] will lead to earlier diagnosis and initiation of treatment in pediatric acute respiratory distress syndrome especially in resource-limited country [10].

Rationale

In previous studies done, the criteria used to label pediatric acute respiratory distress syndrome (pARDS) was based on invasive procedure for assessment of oxygenation. Nowadays, OSI is used as a component to define pARDS, which is non-invasive and readily available. By using this criteria we aim to assess magnitude of pARDS in children admitted to pediatric intensive care unit.

## Materials and methods

This was a hospital-based descriptive study conducted at the PICU of Aga Khan University Hospital over a period of one year from July 2017 to June 2018 after the approval from the Institutional Review Board. By using WHO calculator taking prevalence of pediatric acute respiratory distress syndrome as 66.4% margin of error equal to 7.6%, the calculated sample size will be as 150 [3].

Operational definitions

OSI: Fraction of inspired oxygen concentration x mean airway pressure/ pulse oximetry saturation

ARDS: Acute onset (within seven days of onset) of symptoms like respiratory rate > 40, bluish discoloration of skin and mucous membranes and shortness of breath with bilateral infiltrates on chest radiography consistent with acute parenchymal disease, presence of generalized edema and oxygen saturation index > 5.0, assisted by probe attaching to finger and shown on monitor and oxygen via face mask or nasal cannula [6].

The demographic data collected included age, gender, weight of child and oxygenation status at the time of admission. Patient was examined for shortness of breath, bluish discoloration of skin and mucous membranes. Respiratory rate was measured by counting the number of breaths per minute. History of onset of symptoms was asked from parents and guardians. X-ray chest, anterior posterior view was done and reviewed by radiologist for presence of infiltrates. Probe was attached, monitor was connected and oxygen support was given via face mask or nasal cannula and OSI was measured.

By using SPSS version 22 (IBM Corp, Armonk, NY) data was analyzed. Mean ± standard deviation was calculated for age, weight of child, OSI, duration of symptoms. Frequency and percentages were calculated for gender, bilateral chest infiltrates and edema.

Effect modifiers like age, gender, duration of symptoms, weight of child, bluish discoloration of skin and mucous membranes and OSI > 5 were controlled through stratification. Post stratification chi-square test was applied. P-valve ≤ 0.05 was taken as significant.

## Results

**Figure 1:**
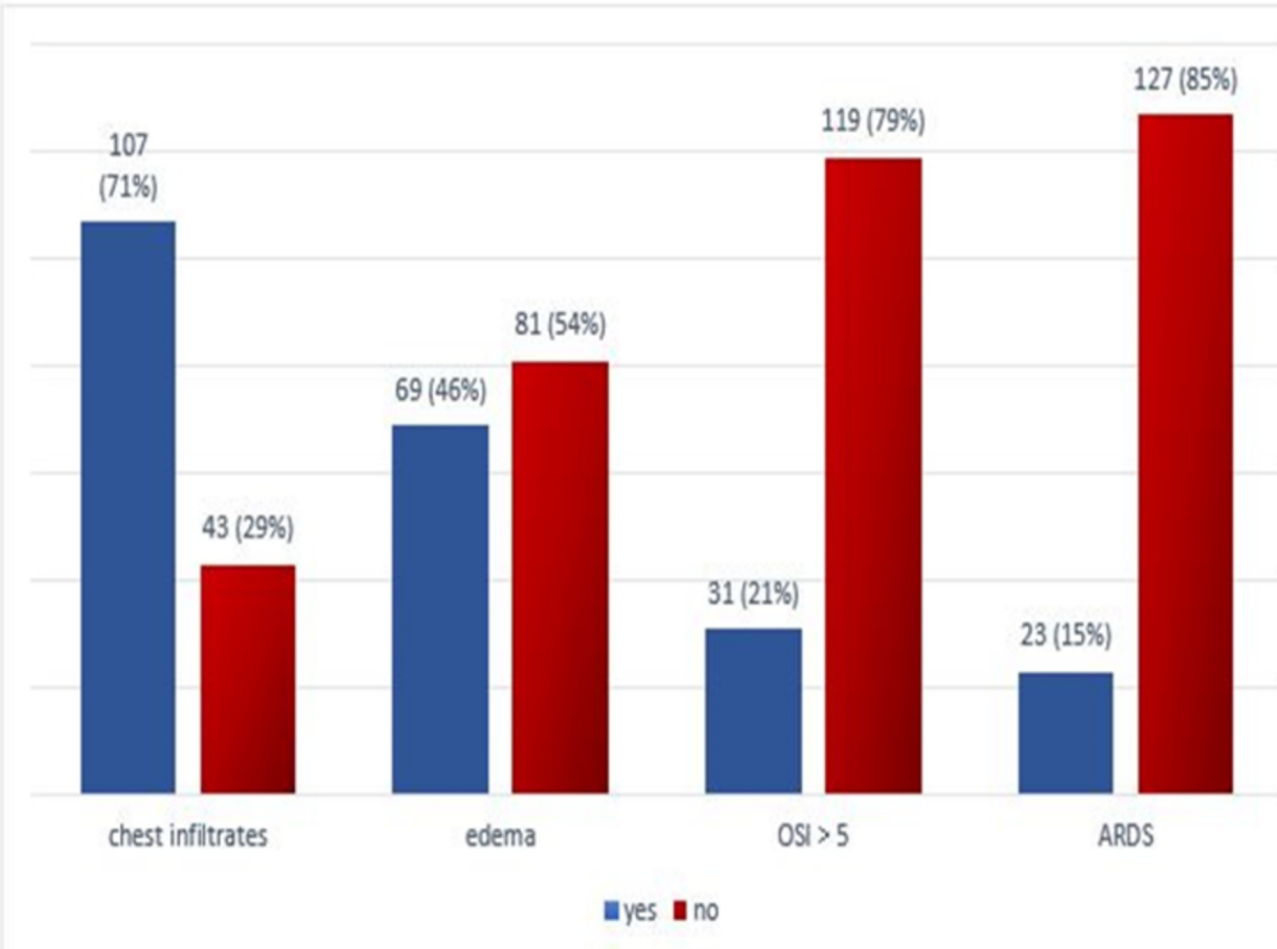
Frequency of edema, radiographic findings and ARDS ARDS: Acute respiratory distress syndrome; OSI: Oxygen saturation index.

Age range in this study was from one month to 16 years with mean age of 38.27 ± 53.13 months. Out of the 150 patients, 92 (61.33%) were males and 58 (38.67%) were females with male to female ratio of 1.6:1. Mean duration of symptoms was 1.23 ± 0.42 days and mean weight of children was 14.53 ± 16.64 kg. Distribution of patients according to bilateral chest infiltrates, edema, OSI > 5 and bluish discoloration of skin and mucous membranes is shown in Figure 1.

In this study, I have found the frequency of pediatric acute respiratory distress syndrome as per new definition in children admitted in a pediatric ICU in 23 (15.33%) patients (Figure 1).

**Figure 2:**
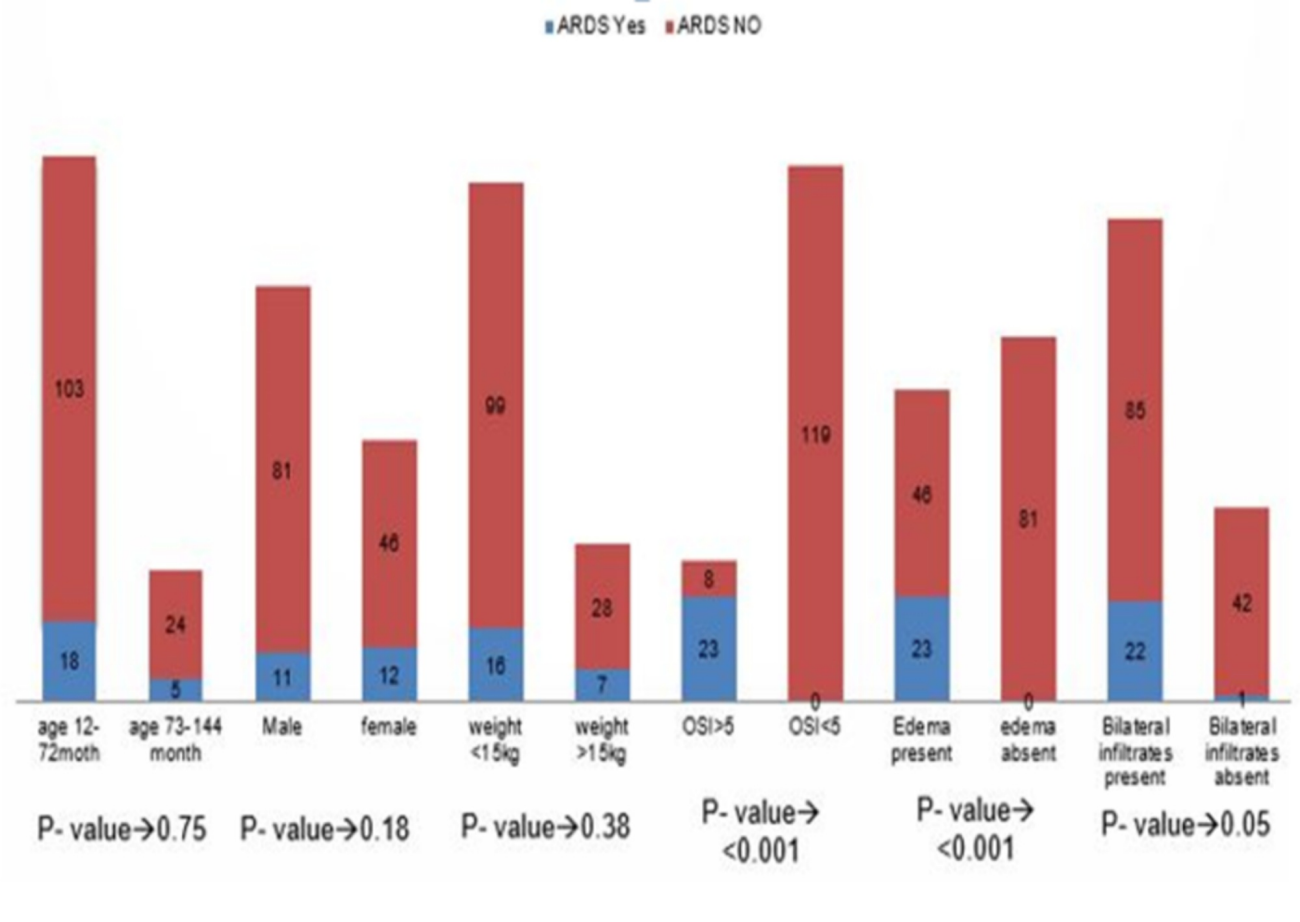
Stratification of pARDS with respect to age, gender, weight, OSI, edema and radiological findings pARDS: Pediatric acute respiratory distress syndrome; OSI: Oxygen saturation index.

Stratification of pediatric acute respiratory distress syndrome with respect to age and gender along with duration of symptoms and weight of baby is shown in Figure 2, respectively. Stratification of pediatric acute respiratory distress syndrome with respect to bilateral chest infiltrates, edema, OSI > 5 and bluish discoloration of skin and mucous membranes is also shown in Figure 2.

## Discussion

Although the epidemiology of ARDS has been well documented for adults, few epidemiological studies have been conducted with children. Studies that used the American-European Conference Consensus (AECC) definition have shown prevalence from 0.86% to 7.8% of PICU admissions, and 5% to 20.5% of ventilated patients [11-14]. Mortality rates ranged from 14% to 61% [4, 14]. Higher mortality rates have been reported in developing countries [11,15]. Despite the fact that the data used in the Berlin study did not include pediatric patients, its validity for infants and toddlers has recently been demonstrated in a study conducted by the European Society for Pediatric and Neonatal Intensive Care [14]. I have conducted this study to determine the frequency of pediatric acute respiratory distress syndrome as per new definition in children admitted in a pediatric ICU of a tertiary-care university hospital.

Age range in this study was from one month to 16 years with mean age of 38.27 ± 53.13 months. Out of the 150 patients, 92 (61.33%) were males and 58 (38.67%) were females with male to female ratio of 1.6:1. In this study, I have found the frequency of pediatric acute respiratory distress syndrome as per new definition in children admitted in a pediatric ICU in 23 (15.33%) patients. A recent study from India reported pARDS incidence from 8.5 to 27 cases per 1000 PICU admissions [2]. Another study from Iran reports incidence from 5 to 10% of total PICU admissions [8].

The incidence of pediatric ARDS is different than adult and it is relatively rare but it is also underdiagnosed due to lack of specific guidelines. Its prevalence in children in the United States, Europe and Australia is 2-12.8 cases/100,000 people per year [16]. In North America, multi-center study reported that 1-4% of children undergoing mechanical ventilation had ARDS among children hospitalized in PICU [17].

A research by Zimmerman et al. was conducted as a population-based prospective cohort study that was designed to determine the population incidence and outcome of pediatric acute lung injury (ALI) [15]. The research was performed at all hospitals admitting critically-ill children in King County Washington. It was done on children aged six months to 15 years who required invasive (through endotracheal tube or tracheostomy) or non-invasive (through a full-face mask) mechanical ventilation. They utilized the AECC criteria. They found a prevalence of 27.6% (39/141) for both ALI and ARDS. Furthermore, ALI accounted for 7% (10/141) while ARDS accounted for 20.6%.

Quartin et al. conducted a prospective cross-sectional study on the prevalence of ALI and ARDS outside the intensive care units [18]. This group evaluated all the adult patients (above 18 years) who were admitted to respiratory isolation rooms on the general wards of a large tertiary hospital over a period of one year. This group utilized the AECC criteria to diagnose the patients. A total of 715 patients were screened, 62 of which fulfilled the ALI screening criteria with a prevalence of 9%. Further, 15 out of 715 fulfilled the ARDS criteria giving a prevalence of 2%.

In comparison, Hughes et al. conducted a prospective cross-sectional study on the prevalence of ARDS in 23 adult intensive care units in Scotland over a period of eight months [19]. A total of 4530 patients aged 15 years and above were recruited into the study. The patients with ARDS were identified using the diagnostic criteria defined by the AECC. About 367 patients were diagnosed with ARDS, giving a prevalence of 8.1%. This study did not assess for patients with ALI. Research by Rubenfeld et al. was conducted on a prospective cohort study in 21 intensive care units in Washington over a period of one year. The group applied the AECC criteria to diagnose the patients. They evaluated mechanically ventilated patients aged 15-80 years. A total of 4251 patients were enrolled. The prevalence of ALI was 26.2% (1113 out of 4251) and that of ARDS was 19.5% (828 out of 4251) [20].

This study has shown that the frequency of pediatric acute respiratory distress syndrome as per new definition in children admitted in a pediatric ICU is quite high. So, we recommend that this non-invasive and readily available method of OSI should be used as a routine for early diagnosis and management of pediatric acute respiratory distress syndrome.

## Conclusions

Our study done in PICU shows that by using non-invasive OSI, early diagnosis of pediatric acute respiratory distress can be made and this will help in better management and less mortality.
